# Acceptability of shared medication coordination in social psychiatric residence consultations: a qualitative interview study

**DOI:** 10.1186/s12888-025-07175-7

**Published:** 2025-09-25

**Authors:** Tina Birkeskov Axelsen, Charlotte Arp Sørensen, Anders Lindelof, Mette Spliid Ludvigsen

**Affiliations:** 1 Hospital Pharmacy, Central Denmark Region, Palle Juul-Jensens Boulevard 240, Aarhus N, 8200 Denmark; 2 Regional Psychiatry Randers, Central Denmark Region, Randers, Denmark; 3 Department of Clinical Medicine, Randers Regional Hospital, Aarhus University, Aarhus, Denmark; 4 Faculty of Nursing and Health Sciences, Nord University, Bodø, Norway

**Keywords:** Shared decision-making, Patient involvement, Medication coordination, Acceptability, Social psychiatric residence

## Abstract

**Background:**

When various physicians prescribe medications to a single patient, insufficient medication coordination (MedCo) may lead to drug interactions and side effects, especially in patients living with severe mental disorders such as schizophrenia. A Danish social psychiatric residence has addressed this issue with an annual MedCo programme based on shared decision-making, involving residents in shared residence consultations and organised MedCo (The *Shared MedCo *intervention). Nevertheless, despite more than a decade in operation, challenges remain in transferring this intervention to other residences, and the underlying reasons for its limited acceptability remain unclear. Consequently, this study aimed to investigate the acceptability of shared MedCo for residents treated under the shared responsibility by multiple physicians and involving residents, residence carer staff, GPs, psychiatrists, pharmacists and decision-makers.

**Methods:**

We employed individual qualitative semi-structured in-depth interviews. The investigation was guided by the seven constructs of the ‘*Theoretical Framework of Acceptability’* and analysed based on Malterud’s ‘*Systematic text condensation’*.

**Results:**

A total of 43 interviews were conducted from August to December 2022. Less significant acceptability constructs were linked to ethicality, intervention coherence and perceived effectiveness. More significant acceptability constructs were associated with affective attitude, burden, opportunity costs and self-efficacy. Key barriers included the siloed nature of healthcare, medication change, resident involvement, inadequate performance support, professional vs. layman language, geographic distances and time-consuming activities.

Key facilitators included consultations hosted and managed in the residence, leader support, supported resident involvement, organised coordination activities, pharmacist and carer staff contributions, sufficient time, clinical routine, and job-satisfaction. Further facilitators were defined roles, expectations and procedures, and feelings of relatedness, security, trust, hope and meaningfulness.

**Conclusion:**

Various barriers and facilitators influence participants' acceptance of shared MedCo. Understanding these factors can assist future implementers in effectively accommodating the implementation of *shared MedCo*.

**Supplementary Information:**

The online version contains supplementary material available at 10.1186/s12888-025-07175-7.

## Background

Mental disorders represent nearly 25% of the global disease burden, exceeding cancer and circulatory diseases [1]. In 2019, the World Health Organization (WHO) reported that 64 million individuals were living with severe mental disorders globally [2]. In this study, ‘severe mental disorders’ refers to psychiatric conditions that cause a clinical disturbance in an individual’s cognition, emotional regulation or behaviour, severely impairing daily functioning (i.e. schizophrenia and bipolar disorder) [2]. People living with severe mental disorder consults their general practitioner (GP) 2.5 times more frequently and experiences twice as many hospitalisations as the general population. Furthermore, they have a higher incidence of deaths from treatable conditions than the general population [3, 4]. Thus, the need for action on mental health was found indisputable and urgent [5]. Numerous treatment options exist for this group [6]. Medical treatment is a crucial component of primary care in both the acute and maintenance phases of mental disorders [7]. However, challenges persist, including elevated hospitalisation and mortality rates [8–12]. Issues include e.g. drug interactions between antipsychotics and somatic drugs, antipsychotic side effects like diabetes, hypertension and cognitive impairment [13–15], and concurrent use of multiple antipsychotics without discernible benefits [16, 17]. Additionally, due to the high prevalence of multimorbidity in this group [15], the simultaneous use of various medications (polypharmacy) is frequently associated with a lower quality of life [18] and a life expectancy 15–20 years shorter than that of the general population [19–21].

Multimorbidity and polypharmacy complicate patients’ overall healthcare coordination [22]. Healthcare coordination, including medication coordination (MedCo), ensures proper care and medication management by organising resources and facilitating communication and information exchange among healthcare professionals (HCPs) who share patient care responsibilities [22, 23]. Promoting the exchange of medication information between multiple practitioners affiliated in different contexts of society (e.g., a GP in primary healthcare and a psychiatrist in secondary healthcare) poses a particular challenge for individuals who rely heavily on extensive support networks. A solution can be for them to live in a social psychiatric residence (residence). Staffed by a variety of professionals and caregiver employees (carer staff), these residences play a crucial role in caring for the patients (from now residents) [24] as carer staff now may be responsible for communication and information exchange between HCPs and the residents to support MedCo [25].

In the context of residential care, legal obligations exist. Legal medication obligations may include annual healthcare assessments by the respectively practitioners [26–28] and resident evaluations covering general health and medical treatments [29]. Insufficient formal communication and collaboration between HCPs internally and with carer staff may cause task overlapping, which is time-consuming and may create a disjointed and stressful MedCo process for residents, carer staff and HCPs alike. Therefore, streamlining and organising MedCo among all parties involved in the resident is important to reduce challenges and ensure an efficient and less stressful care process [30, 31]. Studies emphasise the importance of organising MedCo between activities in the context of healthcare setting outside the residence (e.g. prescribing, deprescribing, assessing medicine in the GP clinic or by the psychiatrist in the hospital) and activities in the social living setting inside the residence (e.g. dispensing, administrating, consuming, assessing side-effect) [31–42]. As for the healthcare setting, clinical pharmacists may coordinate and improve the quality of medication treatment, facilitate medication discussions among HCPs, reduce antipsychotic polypharmacy, reduce hospital visits and length of stay, and improve mental health outcomes [31, 39, 42–44]. As for the social living setting, nurse coordinators may promote patient coherence, facilitate communication between HCPs and patients/residents, and enhance participant involvement and motivation [40, 41, 45]. Additionally, research shows that efforts to address MedCo challenges can include shared decision-making (SDM) and patient involvement (PI), e.g. ensuring that HCPs and patients reach a shared clinical decision based on the best available evidence and the patient’s preferences, respecting the patient’s autonomy [46].

*SDM* and in-person, cross-professional collaboration are vital [30, 31], feasible in psychiatric healthcare [47] and advocated as the best decision-making model to achieve agreement between HCPs and patients regarding long-term treatment goals [48]. Furthermore, SDM improves relationships, aligns decisions with patients’ needs, reduces repeat consultations, enhances patient satisfaction [49] and counters contextual, siloed thinking [50]. Recognising all participants, including residents as an assumed part of the SDM process and experts in their own field, fosters an improved decision-making process, minimises conflicts [51, 52] and generates solutions transcending individual capabilities [53, 54].

*PI* enhances medical treatments, helps avoid misunderstandings [55–59], improves individuals’ influence on their care decisions [60], improves participant satisfaction and allows for integration between the context of the healthcare setting (as for HCPs) and the social living setting in the residence (as for carer staff and residents) [61]. Also, PI leads to shared decisions better aligned with the individual patient, greater patient satisfaction, fewer repeat consultations [49] and improved patient outcomes [62], which may reduce medication adjustment harms through collaborative planning, tapering and monitoring [63]. Thereby, PI is an assumed part of the SDM process. However, patients/residents diagnosed with severe mental disorders are associated with some degree of cognitive disability, which may affect their participation ability. Still, most people diagnosed with a severe mental disorder prefer to be involved in decision-making [64]. Therefore, special efforts are needed to approach PI in the SDM process. Additionally, carer staff need to communicate resident concerns to HCPs [62], and managers need to empower carer staff voices [65].

Experience with an SDM, PI and organised MedCo (defined as MedCo in the community, in the residence and during shared consultations) intervention has been collected for more than a decade in a residence in Denmark (the original residence) [33, 66]. This shared MedCo intervention is performed as an annual 15–20 min shared residence consultation between a GP, a psychiatrist, a clinical pharmacist, a nurse/healthcare coordinator, carer staff and the residents while engaging and supporting the resident in the process.

These shared residence consultations were implemented after a health investigation (2008), revealed widespread medication use and poor health among the residents [67]. The investigation led GPs, psychiatrists, psychiatric nurses, residence managers and carer staff, to address the key issues by establishing the annual coordination approach. The intervention assigned tasks to each participant: the GP did health checks, the psychiatrist handled psychiatric assessments and the staff managed logistics and coordinated PI. PI was approached by carer staff, who, over the course of a month, supported the resident in completing a comprehensive health and medication questionnaire, the results of which were discussed during the shared residence consultation. This model was slightly contextually adapted over the years, e.g. by engaging a clinical pharmacist who conducted pharmacist-led medication review and participated in the shared consultation [68]. In addition to time savings and coordinated treatment plans, the benefits of this approach include earlier somatic disease detection, reduced medication use and interactions, decreased medication side effects, lower healthcare use and costs, improved quality of life for residents and increased job satisfaction for carer staff [33, 66].

However, despite political attention [69–72], several (unpublished) attempts to transfer the shared MedCo intervention to other residential contexts have shown limited success [32]. Understanding the barriers and facilitators of acceptance of shared MedCo is essential to developing a shared MedCo intervention that is widely accepted. However, this precondition to successful implementation [51, 52, 73, 74] has yet to be fully investigated.

### Aim

The aim of this study was to investigate the acceptability of shared MedCo for residents treated under the shared responsibility by multiple physicians and involving residents, residence carer staff, GPs, psychiatrists, clinical pharmacists and decision-makers.

## Methods

### Design

This study is a subset of a larger project aimed at developing and adapting an evidence-informed, multi-stakeholder SDM and PI model for a residential integrated healthcare and social living MedCo intervention. This project is guided by *The framework for developing and evaluating complex interventions in healthcare* from the UK Medical Research Council (MRC). The MRC framework states the importance of addressing acceptability as a prerequisite to intervention adherence [75] during the initial development of an intervention [76, 77].

To improve delivery, ensure transferability, enhance our understanding of shared MedCo and for promoting participant acceptance in future implementations [78], the present study employed a qualitative research design using individual semi-structured, in-depth interviews to investigate participants’ acceptance of shared MedCo across multiple levels, i.e. residents, carer staff, HCPs, decision-makers and other stakeholders [79]. *The theoretical framework of acceptability (TFA)* was applied to guide the development of an interview guide and inform the analysis and interpretation of findings based on seven constructs: affective attitude, burden, ethicality, intervention coherence, opportunity costs, perceived effectiveness and self-efficacy [80].

The interviewer was the primary author and a trained clinical pharmacist with experience in pharmacist-led residential medication reviews in the original residence [81, 82], thus having prior knowledge of most decision-makers and HCPs within this study. However, there was no affiliation with the study sites or any professional relationships or responsibilities with the participants during the study period.

To ensure complete and transparent reporting, we were guided by *The consolidated criteria for reporting qualitative research* (COREQ) [83] (See supplementary File 1).

### Setting and participants

The study was conducted in a social psychiatric residential (residence) *setting* in three out of five regions in Denmark. Danish residences accommodate patients living with severe mental disorders such as schizophrenia or bipolar disorder, somatic co-morbidity and drug or alcohol abuse, who additionally face significant challenges in daily functioning, with reduced social function, cognitive disabilities, and with an affected score on the Global Assessment of Functioning Scale (GAF). GAF is a clinician-rated scale that rates individual persons living with mental disorders for symptom and functioning severity on a scale from 100 (extremely high functioning) to 1 (severe impairment) [84]. The residents are referred from their local municipalities and require long-term treatment and support, which is not available in primary or secondary care settings. They may (initially) present with severe or uncontrolled conditions and may lack the ability to manage their self-care or medication independently, thereby necessitating close monitoring and assistance from the carer staff. The original residence provided 24-hour care for adults living with severe mental disorders, somatic illnesses (often including Korsakoff’s psychosis), challenging behaviours, legal issues and a GAF score below 40, all of whom required specialised support for impaired social functioning [33, 66].

*Participants* were purposively sampled to address the research aim, ensuring rigour, trustworthiness and reliability of the data and findings [85]. Purposive sampling provided an adequate representation and variation of perspectives from the intervention developers, the users and the future users. Additionally, we sought to include residents, carer staff, HCPs and decision-makers incl. other stakeholders which provided an adequate variation and representation of shared MedCo experience.

Participants eligible for inclusion met the following criteria: (i) Residents aged more than 18 years who were prescribed medication by both a GP and a psychiatrist. Due to their mental disorder and to ensure adequate representation of their perspectives, residents were allowed support during the interviews, i.e. (minimal) assistance by a carer staff was allowed. (ii) Carer staff (nurses, social and healthcare assistants, occupational and physical therapists, mental health support workers) employed to care for the residents. (iii) HCPs (GPs, psychiatrists, clinical pharmacists and clinical pharmacologists) affiliated with the eligible residents. (iv) Decision-makers and other stakeholders (policymakers, resident association representatives, managers, senior psychiatrist consultants and administrative staff).

Participant experience spanned from *extensive experience*, i.e. with several years of experience with shared MedCo as performed in the original residence (to understand implementing and performing shared MedCo). S*ome experience*, i.e. participants experienced with partly successful attempts to implement the shared MedCo model (to understand the experience of former implementation attempts). *No experience*, i.e., participants with no prior experience with any shared MedCo implementation activities (to understand what participants anticipated before implementing the intervention).

HCPs, decision-makers and other stakeholders were identified and invited by the primary author based on prior field experience. Following the inclusion criteria and guidance from the primary author, residence managers invited suitable carer staff. Resident participants were identified and invited by the carer staff, who applied their knowledge of their residents to this process. Residents who were unable to give informed consent were excluded.

### Data collection

Interviews were conducted one-on-one in Danish language, either in-person or online/telephone, and at the participants’ preferred time and location. All participants were given time to consider their participation. Written informed consent was obtained either by hand or by e-mail before the interview. No compensation was provided.

A semi-structured interview guide with open-ended questions was developed, inspired by qualitative interview methodology and TFA [79, 80]. The interview guide was qualified by representatives across participant levels, i.e. residents, carer staff, HCPs, decision-makers/stakeholders (Supplementary File 2). A pilot test of the interview guide was performed with an experienced psychiatrist. To address residents’ cognitive impairments, a revised interview guide was developed for this group. This interview guide was qualified by residents and carer staff without pilot testing (Supplementary File 3).

The interviews started with an open-ended question: ‘*In Denmark*,* medication responsibility can be shared between a GP and a psychiatrist. What is your experience with this?.*’ The dialogue persisted based on participants’ responses. The interview continued with questions from the interview guide, which, based on the TFA constructs, addressed the research aim. For example, ‘*How did this affect your feelings?*’ (Affective attitude). ‘*How big an effort did you have to make to participate in the shared residence consultation?’* (Burden). ‘*How does the residence model match your personal and moral core values?*’ (Ethicality). *‘What is/do you think the main purpose of the residence model is?*’ (Intervention coherence). *‘How does the residence model live up to this main purpose?*’ (Perceived effectiveness). ‘*What benefits*,* profits*,* values did/do you have to do without?’* (Opportunity costs*). ‘Do you feel confident that you could/can display the behaviour required to participate in these shared residence consultations?’* (Self-efficacy).

Interviews were audio-recorded, with recordings accompanied by field notes and ongoing pre- and post-interview reflections. Additionally, the interviews were transcribed verbatim in Danish by a research assistant using the ‘KONCH’ audio transcription programme [86].

The authors continuously assessed participant inclusion, guided by the principle of representativity and variation and concluded when adequate data of sufficient quality and depth had been collected to gain information power enough to address the study aim [87].

### Data analysis

**Fig. 1:**
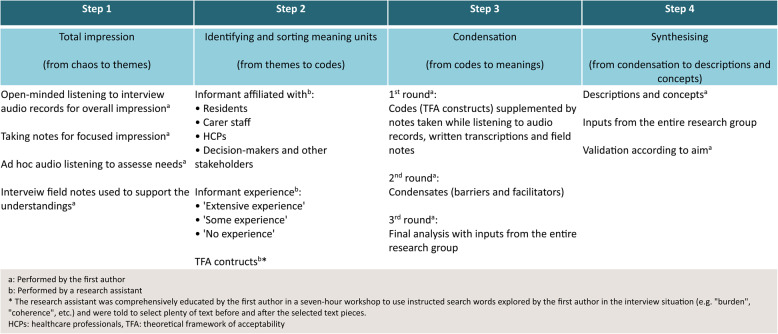
Data coding process

The analysis of interview data was conducted in Danish and inspired by methodologies outlined by thorough listening to audio recordings and Malterud’s approach as presented in “*Systematic Text Condensation”*, employing manual coding, analysis and thematic text condensation [88]. Data codes were guided by the seven TFA constructs [80]. The data coding process is outlined in Fig. 1.

## Results

Interviews were conducted between August and December 2022 and lasted between 20 and 125 min, averaging 62 min. No interviews were repeated, transcripts were not returned to participants for comments or corrections, and no participants provided feedback on the findings. The pilot interview produced only minor corrections of the interview guide and was included in the study.

A total of 47 individuals with varied backgrounds and experiences with shared MedCo were invited, and 43 accepted the invitation to participate in the interviews before enough information power was gained to answer the research aim. One decision-maker, one HCP and one carer staff could not make time for the interview, and one HCP failed to respond to the invitation. The participant characteristics are illustrated in Table 1.

**Table 1:** Participant characteristics (*n*=43)

		**Extensive experience**	Some experience	**No experience**	**Total **
Age/year	< 30				**1**
	30-50				**34**
	>50				**8**
Sex	Men	9	0	9	**18**
	Women	11	12	2	**25**
Participant level	Decision-makers and other stakeholders	6	2	4	**12**
	Healthcare professionals	7	8	1	**16**
	Residence carer staffs	4	2	1	**7**
	Residents (patients)	3	0	5	**8**
Total		**20**	**12**	**11**	

Acceptability key findings are outlined below and arranged according to the seven TFA constructs [80].

Varying degrees of detail are provided for each construct due to the diversity of our findings. Some constructs are purposefully presented specifically for each participant level and experience level, whereas others are presented as aggregates. Thereby, affective attitude, burden, opportunity cost and self-efficacy are presented in detail, as these constructs emerged as the most significant. Ethicality and intervention coherence/perceived effectiveness are covered more briefly.

The main barriers to and facilitators of acceptability of shared MedCo are illustrated in Table 2 and detailed below. Supplementary File 4 provide a comprehensive review of barriers and facilitators of acceptability of shared MedCo affiliated to participant level and experience.

**Table 2:** Main barriers to, and facilitators of acceptability of shared MedCo

**TFA construct**	**Barriers**	**Facilitators**
Affective attitude	Stressful shared consultations	Relationships and feelings of relatedness Feeling of hope, trust, security, pride, respect, fairness, meaningfulness and justice Job satisfaction
Burden	Siloed nature of healthcare Imaging time-consuming approach Establishing HCP collaboration Professional vs. layman language Concern about involving residents in decisions Concerns about medication changes Doubt about PI Scepticism regarding performance possibilities Unclear role, task descriptions and expectations Negotiations between the municipalities and residences Municipal funding residential activities	Sufficient leader support and prioritisation of interventions Allocating sufficient time Courageously collaborative adjusting residents' medications Experience Coordinating in advance performed activities Release of information exchange responsibility Health-educated carer staff Residence hosting and management of logistical and PI activities Collaboration Defined roles, expectations and procedures Comfortability Pharmacists counteracting physicians'siloed affiliations Simultaneously performing SDM in shared residence consultation
Ethicality		Resident-centred healthcare and residents as equal partners Respecting residents’ voices Equal opportunities
Intervention coherence/Perceived effectiveness	Varying ‘main purposes’ of the intervention	Individual ‘main purposes’ fulfilled for all participants
Opportunity cost	Time-consuming implementation and performance Uncompensated consultations New practice for approaching consultations	Effort without scarifying quality Worthwhile time and energy investment in the long run Simultaneously performing time-saving coordination activities Benefits outweigh the effort and are achieved between consultations Cost-effective
Self-efficacy	Inadequate performance support Professional and personal concerns Lack of carer staff confidence	Leader encouragement Carer staff supporting the resident’s voice Coziness and coffee

Condensation of findings expressed by different participant levels (residents, carer staff, HCPs, decision-makers) and with different shared MedCo experience levels (extensive, some, no experience) (*n*=43)

*PI* Patient involvement, *SDM* Shared Decision-making

### Affective attitude

Affective attitudes concern *how an individual feels about the shared MedCo intervention*. Overall, participants across varying levels and experience expressed positive perceptions on the concept of shared MedCo. Participants across all levels and with extensive experience mainly reported positive feelings, while participants across all levels with no experience mostly noted negative feelings. The main negative feelings expressed were apprehension about changing residents’ medications and involving them in decisions. This was not an issue for the experienced participants.

*Residents* with extensive experience found shared residence consultations to be stressful.

However, they also experienced feelings of hope and what was interpreted as trust and security. The residents elaborated:

*‘I remember at first*,* I wasn’t happy about it*,* but I am now - they know something about me and I can also speak.’ (Resident 28)*.

*‘You use energy to keep an eye on yourself – sharing (the worries) isn’t all happiness. Now*,* I hand over the responsibility to them – I’m in good hands.’ (Resident 30)*.

This resident explained:

*‘… because if I participate*,* there’s like a hope*,* and if I’m not participating*,* I feel like there is no hope. Then I throw it away.’ (Resident 24)*.

Residents valued the opportunity to speak with both their GP and a psychiatrist simultaneously, noting that carer staff support helped them manage consultation stress.

*‘Then*,* I have to talk about myself to complete strangers*,* and it’s a bit stressful. So*,* it helps that they (carer staff) come along… I want to participate because I want help.’ (Resident 44)*.

Residents without cognitive impairment or shared MedCo experience envisioned that the benefits from participating in shared MedCo would outweigh the efforts compared to the current responsibility to exchange information between multiple practitioners.

*‘I feel that it (information exchange) puts a lot of pressure on me. It (shared MedCo) would help my psyche a lot, and it would probably also reduce some of my emergency admissions.’ (Resident 21)*

*Carer staff *described how the interdisciplinary collaboration fostered a sense of safety, securety and justice and that they felt proud experiencing trust, fairness and job satisfaction from giving residents a voice and building strong relationships with them and their HCPs. This carer staff elaborated:

*‘Shared consultations bring together the different specialities, so that we trust that we’re doing the right thing. It offers a sense of safety and security, knowing that people have actually addressed this in a qualified way.’ (Carer staff 29)*

And supplemented:

*‘I feel that it (MedCo) somehow gives some justice, because the residents are given opportunities and possibilities that are tailored to the difficulties and limitations that they face. I don't think we could do without it. It wouldn't be fair.’ (Carer staff 29)*

Additionally, in difficult situations – such as with residents displaying aggressive behaviour, for example, as a result of medication deprescribing – the feeling of safely being supported by known and trusted HCPs was described as essential:

*‘You feel safe knowing that the doctors have your back, and that means we can stay longer in the situation (e.g. with aggressive residents)…’ (Carer staff 18)*

*HCPs* emphasised feelings interpreted as meaningfulness, professional pride, security and feelings of relatedness. One GP elaborated on shared MedCo as a contrast to the feelings of insecurity when they were solely responsible for residents' medications.

*‘But it (solely responsible) doesn't always make me feel completely comfortable. And if somatic medicine is then combined with…. an antipsychotic, then I'm screwed, I am. But then you try to do it the best you possibly can. But you're not always professionally competent to do that. You are competent enough, but not as much as you would like. Then, you feel inadequate, and that's annoying. But I live with that too.’ (GP 14)*

GPs with extensive experience elaborated on their feeling of relatedness to the other participants as: *‘Shared MedCo makes sense because it's just different being together in-person around a table’* (GP 31), and another GP described:

*‘I feel really good about it (shared MedCo). I think it's nice to feel that we make a difference... it's certainly not ‘for the money’ (no consultation fee)... I feel pride that we make a difference for some patients here who actually have a hard time on many parameters.' (GP 34)*

*Decision-makers *expressed pride in connecting healthcare and social living settings as seen in the following*: ‘It’s something we should stick to because it’s really, really important for those people who have the hardest time in our society.’ (Decision maker 12).*

Additionally, this experienced stakeholder explored pride and satisfaction with the reduced medication consumption:

*‘The point where I really took shared MedCo to my heart was during the first analyses, and I saw that there were many residents where we could reduce their medication use. Shared MedCo makes me proud and happy.’ (Stakeholder 20)*

### Burden

Burden refers to *the perceived or anticipated amount of effort that is required to participate in the shared MedCo intervention*.

Participants across all levels and experience noted that aspects associated with the siloed nature of the healthcare system – characterised by multi-disciplinary treatments with no formal cross-professional communication or collaboration – posed a burden to shared MedCo. However, at all experience levels, participants reported that factors such as leadership and prioritisation, carer staff support, clear roles, a clear systematic approach to performance and allocation of sufficient time helped mitigate barriers.

The degree of experience with shared MedCo intervention appeared to significantly influence how participants perceived shared MedCo burden. In particular, the time aspect was prominent. While participants with no shared MedCo experience viewed the intervention as: *‘A time-consuming burden for the participants…’(Psychiatrist 13)(GP 07)*, those with experience expressed that: ‘*Not much time is spent on it - in the grand scheme of a year’s work*,* you get a lot in a short amount of time’ (Psychiatrist 39).* Further quotes along the same lines: *‘It saves time and makes things easier and faster’ (GP 31)*, and ‘W*e’re already spending time on it*,* now it’s just coordinated - no doubt it’s worth the time and energy.’ (Carer staff 29).*

*Residents* without cognitive impairment and no shared MedCo experience envisioned a reduction in burden by making decisions jointly, rather than bearing the responsibility of navigating between two separate providers. One resident stated:

*‘It would be really good if all three of us (GP*,* psychiatrist*,* resident) could sit down and figure out what to do with my medication*,* instead of me having to be in multiple places at once*,* which is really*,* really difficult.’ (Resident 21)*.

*Carer staff* with no shared MedCo experience expected that efforts would go into establishing the intervention. Particularly the parts involving HCPs, due to, e.g., the use of professional language rather than layman’s terms, were mentioned. One carer staff shared that:

*‘Getting a good dialogue with the physician requires an effort. Some residents…*,* we can’t just take them to the GP and if the GP also seems nervous and uneasy around them*,* it makes things really difficult.’ (Carer staff 16)*.

Health-educated residence carer staff (nurses or social and healthcare assistants) and the residence hosting the intervention were considered part of the practical solution. One carer staff (RN) elaborated on what she was told and experienced after her employment in a residence:

*‘Back then (before nurse employment)*,* it was very difficult for the staff to collaborate with the GP and the psychiatrist because they just didn’t speak the same “language”. Now*,* I try to speak both healthcare and resident languages and being the one who coordinates the dialogue. Therefore*,* we now have a much better collaboration.’ (Carer staff 18)*.

Carer staff with shared MedCo experience repeatedly highlighted that having consultations hosted and coordinated within the residence mitigates the performance burden:

*‘It’s important that it’s an internal person (within the residence) who coordinate the process—so that the resident gets the best out of the time spent at the consultation. We (the residence staff) have to saddle up to make things easy for the HCP; we have to take the lead.’ (Carer staff 29)*.

Supplementary, *Carer staff* and *HCPs* with no experience expressed concern about the burden of involving residents in decisions and about changing their medication. A GP noted:

*‘There might be aggressive*,* withdrawn*,* or brain-damaged patients. If there’s no resident communication*,* it leaves you (GP) reliant on observations and measurements*,* and that’s difficult.’ (GP 14).*

In contrast, this carer staff with no shared MedCo experience, despite anticipating that patient involvement would be demanding, shared the following expectations about how it might work in practice:

*‘Well*,* I think I have to appear and be the resident’s supporter or*,* if necessary*,* his advocate. After all*,* I’m the one who knows the resident*,* so I’ll have to prepare myself so that I’m as ready to talk about how he’s been doing as if it were me telling the HCP how I’m doing.’ (Carer staff 16)*.

Medical treatment optimisation was an underpinning concern. Complex medication regimens made HCPs concerned about solely adjusting treatments:

*‘I don’t know what medicine works for what. I don’t know what is a side effect and what was given for side effects… So*,* it can be very difficult.’ (GP 14)*.

The increased workload on GPs was also explored: ‘*The more medication changes one would agree upon at such a consultation*,* the more tasks the GP will have afterwards*.’ *(Pharmacist 03).*

Doubt and concern about medication changes were reflected in the experienced carer staff’s views. This experienced carer staff highlighted the benefit of courageously and in collaboration adjusting residents’ medications and involving the residents.

*‘Before (shared residence consultations)*,* the residents’ medicine lists were endless*,* and they have really reduced now. So*,* the thing about also having the courage to… “Now we’re trying to change the medicine*,* and if it doesn’t work*,* we’ve tried”. When we adjust their medication*,* we do it together in a well-considered way*,* and the resident is involved. I am fully confident in that. It’s something there is a dialogue about. So*,* it makes a huge difference.’ (Carer staff 29)*.

An experienced carer staff added that improvements in residents’ behaviour following collaborative medication adjustments served as a key motivator for continuing assess their treatment:

*‘There have been some changes in his medication and now it’s extremely rare that we need to use force against him…(pause)… in general*,* if the resident is well medicated*,* they are more balanced*,* and thus easier to support in social activities’. (Carer staff 16).*

A GP emphasised transportation to the residence as burdensome: ‘*I need half an hour’s travel time to the residence and half an hour back home*,* and that takes time from my other patients.’ (GP 07).* Also, the HCPs explored what was needed as a minimum to counteract the burden: *‘What should my role be in a meeting like that…and how do I live up to it?’ . (GP 26).* This is supported by this pharmacist exploring known procedures:

*‘…and that’s also what I’m asking for*,* like “now you know the procedure for this” and “you can look it up here to see what you need to do”’ and now it’s being managed… you know… ‘just like with cancer patients.’ (Pharmacist 02)*.

This was followed by a description of shared residence consultations by a clinical pharmacologist with experience in hospital-based shared consultations:

*‘You have to figure out*,* “what exactly is it that I’m bringing to the table here?“… and you need to focus on “this is my area of expertise in relation to the patient’s overall condition”. And that’s how it has to be for everyone involved in shared consultations… because I think that’s when—well*,* now you have those defined roles*,* and now you actually contribute to something shared.’ (Clinical pharmacologist 11)*.

The benefit of delegating logistical and preparatory tasks to others on-site, i.e. to the residence, was elaborated on by an experienced psychiatrist:

*‘There are others who make sure it will work out. I think that’s super cool. It’s cosy*,* smooth and comfortable. It’s something I look forward to.’ (Psychiatrist 01)*.

Additionally, experienced HCPs emphasised that the presence of the pharmacists is promotes shared MedCo. Beside a coordinated medication list, the pharmacist counteracted physicians’ silo affiliation and the inherent respect for other physicians’ medication prescriptions. This GP elaborated on his gain:

*‘The pharmacist’s advice gives us something to build on when sitting together. The medication lists become more qualified because it’s not guaranteed that we would be able to make it as comprehensive as we can now when the pharmacist is involved.’ (GP 31)*.

Additionally, the clinical pharmacists emphasised that simultaneous SDM eases the burden of delivering their medication review results to the resident’s various HCPs and that the practitioners welcome input from the clinical pharmacist.

*‘I really believe in the pharmacists’ general skills in connection with the comprehensive medication review*,* and that we see different things than the GPs and psychiatrists do. That’s what I see on a daily basis*,* and also that they (practitioners) are really happy with the input the pharmacists provide.‘ (Pharmacist 06)*.

Additionally, the internal relatedness between participants made shared residence consultations and thereby SDM and PI feasible and beneficial. A GP stated that:

*‘Well*,* it’s the fact that it’s a shared consultation where I sit with the psychiatrist*,* and a pharmacist*,* plus the staff… so we’re a team. Everyone has the same focus on trying to get an overview of this resident from whatever angles there may be.’ (GP 31)*.

*Decision-makers* from all experience groups commented on the ongoing burdensome negotiations between the municipality and the residence regarding the economic obligations, which they found were a barrier to shared MedCo. This decision-maker emphasised:

*Where the municipality viewed shared residence consultations as a health professional intervention*,* i.e. a regional obligation*,* the residence perceived it as a social and educational task and a way of supporting the residents in their own healthcare initiatives*,* i.e. a municipal obligation. Since municipalities control the funding*,* and we are only paid for services rendered*,* it’s not simply up to the residence manager to decide to implement shared MedCo. This is extremely troublesome…! (Decision-maker 20)*.

### Ethicality

Ethicality concerns* the extent to which the shared MedCo intervention has a good fit with the individual’s value system.*

All participants at all experience levels unanimously and individually expressed that the overall concept of shared MedCo aligned with their personal and moral values.

*Residents* expressed that: ‘*It might be a small thing for others*,* but it’s a really big thing for me’ (Resident 44)*, and both carer staff and HCPs stated that: ‘*It’s important that we see residents as equals’ (Carer staff 27)*, and: *‘Because it’s their life*,* they must be heard’ (HCP 26).*

Some participants express ethical considerations in more passionate terms. As one decision-maker noted: *‘If you know that psychiatric patients have excess mortality*,* then you have to do something’ (Decision-maker 40)*, and another reflected:

*‘It is the little man*,* fighting against the whole system*,* who has to have a voice too… otherwise*,* we’ll forget the human being*,* and I can’t do that.’ (Stakeholder 32)*.

Similarly, a carer staff stressed: *‘…at least we had to give them the same opportunities as the rest of society have’ (Carer staff 27)*, while a decision-maker underscored urgency: *‘We need to take care of them*,* for God’s sake…!’ (Decision-maker 40).*

One decision-maker noted that shared MedCo aligned with his values by promoting motivation and giving residents a voice:

*‘Well*,* it speaks very strongly into that (ethical values) … It’s about the involvement of the residents and the belief that no matter how miserable you may be*,* there is still a voice in there and there is still a motivation*,* and as long as people have the motivation to breathe*,* then there is motivation. In other words*,* ‘No decision about me without me.’ (Decision-maker 38)*.

### Intervention coherence and perceived effectiveness

Intervention coherence *assesses the extent to which participants understand the shared MedCo intervention and how it works*. Perceived effectiveness refers to *the extent to which the intervention is perceived as likely to achieve its purpose*.

Overall, participants across different levels and experience expressed varying and individual main purposes for shared MedCo. However, all consistently agreed that their individual main purpose would be fulfilled by engaging in shared MedCo. One experienced nurse noted on the multifaceted nature of shared MedCo.

*‘I think the main purpose is to get around the most important health issues that this resident is now exposed to*,* so the whole assessment and coordination of the overall effort. Yes*,* and then to ensure the best possible quality of life for the resident. It’s also really to see if we can get as much information out as possible so that we can provide the best support for the residents… and then coordinate it (the information) … so that we don’t step on each other’s toes. And it can have something to do with social psychiatric approaches… something educational… There could be something with somatic medicine*,* and there could also be something from psychiatry … so the whole assessment and coordination of the overall effort… also it’s really*,* really important that the resident feels secure when we are present in the shared consultation… Hmmm… Basically*,* it’s about many*,* many things!’ . (Nurse 35)*.

### Opportunity costs

Opportunity costs were related to the *extent to which benefits*,* profits or values must be given up to engage in shared MedCo.*

All participants expressed that an effort was made to manage shared residence consultations and resident involvement. However, experienced participants explained that it was worth the time and energy. A resident stated:

*‘Yeah*,* but listen*,* when I’m having a bad day*,* for example*,* I can get really angry and super frustrated about having to go to this consultation. But it usually passes again ((laughs))’ . (Resident 30).*

One experienced carer staff explained:

*‘The (practical) stuff takes up a lot of attention in the month leading up to*,* in the month it runs*,* and the month after (the consultation) - but not in the sense that it’s a burden… it takes up space in a way that makes sense.’ (Carer staff 29)*.

Furthermore, they stated that in the long run, shared MedCo is worthwhile in terms of time and energy, providing benefits without any drawbacks. For example, one carer staff mentioned the reduced burden and time expenditure owing to simultaneous coordinated administrative and regulatory work, and hosting of the residence:

*‘It is definitely not a burdensome effort. These are some areas I already have to deal with. This approach just ensures that it will happen at least once a year —It’s time-saving for me. And now that they (GP*,* psychiatrist*,* pharmacist) are present here at the residence and the resident therefore doesn’t have to leave the house*,* the resident is able to participate and actually has more energy.’ (Carer staff 29)*.

Conversely, *HCPs* with no MedCo experience expressed skepticism about engaging in consultations within a residential context. One GP described the traditional structure of care, where patients are expected to seek help rather than being approached:

*‘My work as a GP has been defined by the patients coming to me with a health problem. I haven’t been the one doing outreach or identifying issues. I’ve tried to help the patients with mental disorders as thoroughly as possible, provided they’ve come to my consultation. I’m actually okay with that because that is what is possible for me to do….’ (GP 31)*.

In addition, despite good intentions, the GPs expressed the issue of being required to deliver additional services and especially of conducting consultations that were not covered by a consultation fee:

*‘General practice is a system where you (the system) add on. So, if you want to add more activities to the system, then you simply have to get more hands on deck. ((Long pause)). Well, you could say that the flippant answer would be that, as it’s the same fee I get for any consultation, then it can be a little difficult not to go for the quick and easy ones.’ (GP 07)*.

In contrast, GPs with experience of shared MedCo unanimously agreed that the benefits outweighed the effort, without compromising quality, and were achieved in the periods between the annual consultations:

*‘Of course, it’s a benefit. Well… now the next consultations are here… now it’s a marathon… But it’s an investment that forms the basis for my remaining days of the year to work much easier… less stressful… while maintaining my professional pride.’ (GP 26)*

*Decision-makers* reported that coordinating cross-system priorities would have long-term economic benefits.

*‘It just became much more systematised… both the health professional part and the social professional part… plus the GP, psychiatrist and pharmacist collaboration as well. So, I’m absolutely certain that it will be much more cost-effective in the end.’ (Decision-maker 33)*.

### Self-efficacy

Self-efficacy pertains to the *participants’ confidence that they could perform the behaviour required to participate in shared MedCo*.

Overall, lack of self-efficacy was not an issue for any participants with extensive experience, although it might have been so in the beginning.

A *resident* discussed managing his insecurities:

*‘It is nerve-wrecking because it’s focused on me*,* but it’s okay. He (psychiatrist) believes in me and sees the potential for my future. I feel like they know me… Cosiness at the meeting is important. They invite me in for coffee. That is nice. I like coffee. So I’m like the main person in the meeting. It’s a free space where you can bring up an issue when something is bothering you*,* or if you’re afraid that some medicine is wrong.’ (Resident 28)*.

Experienced *carer staff* described how supportive leadership initially played a key role in strengthening their self-efficacy. As one carer staff explained:

*‘I had a chat with our team leader where I said, “Maybe I’m a bit nervous. Do you think I can do this?” and she said, “Yes, of course you can do this. If there is anyone who can do it, it’s you.” And then you can only think “Oh okay…. yes!” (smiles).’ (Carer staff 16).*

*HCPs* with some shared MedCo experience expressed their concern and experience before participating in the shared consultation and added their suggested solution:

*‘I was thinking about… “Am I going to say something stupid… pharmacologically” … because I’m not a specialist in this area (somatic)… It requires openness from all parties*,* which also includes the fact that “I” don’t know everything*,* and that’s ok.’ (Psychiatrist 13)*.

Furthermore, stakeholder participants with some experience noted what they perceived as other persons’ insufficient self-efficacy. Especially, they emphasised the importance of giving carer staff the confidence to help voice the residents’ healthcare needs. One stakeholder reflected on the varying levels of carer staff engagement:

*‘Some of the carer staff were both well-prepared and just kind of “on”. But others didn’t say anything at all - they just sat there and were very quiet. Carer staff must be given more confidence and know their role because they actually contribute something really*,* really important*,* like helping the resident to step forward and express: “What’s important to me*,*” etc… You can see that their participation is a huge*,* central piece in this.’ (Stakeholder 02)*.

A pharmacist continued:

*‘This (self-efficacy) can be one of the barriers in implementing and performing shared MedCo*,* and it is a very real issue that we seriously have to deal with in relation to this (shared MedCo).’ (Pharmacist 06)*.

To summarise, our investigation revealed that barriers and facilitators were found in all TFA constructs, and at all participant levels, including residents, carer staff, HCPs, decision-makers and other stakeholders and across all experience levels. The constructs of ethicality, intervention coherence and perceived effectiveness did not seem to be either significant barriers or particular facilitators. By contrast, constructs like affective attitude, burden, opportunity cost and self-efficacy were more likely to constitute significant barriers and facilitators.

## Discussion

Providing MedCo services to residents living with severe mental disorders in a residence is challenging, and the acceptability of a predefined successful solution involving SDM, PI and organised MedCo through shared residence consultations (shared MedCo) has generally been low. This study adds to our understanding of factors influencing the acceptability of shared MedCo and thereby how to transfer the intervention to other residential settings. The study identified key barriers and facilitators impacting acceptance among individuals with severe mental disorders who receive dual treatment from a GP and a psychiatrist, and among affiliated carer staff, HCPs, decision-makers and other relevant stakeholders. We identified several barriers and facilitators related to various experience levels and all seven TFA constructs [80] and found that some constructs were more significant than others.

The discussion below is structured according to the seven TFA constructs [80]. First, the less significant constructs are briefly reflected upon (ethicality, intervention coherence and perceived effectiveness). Next, the more significant constructs are discussed (affective attitude, burden/opportunity cost and self-efficacy).

### Ethicality

Regarding the ethicality construct, the fact that the participants unanimously expressed that the aspects of autonomy, health equity and PI approaches as a human right, often explored as “*No decision about me without me*” [89], contributed to ensuring that the intervention aligned with their personal and moral core values, including their personal core mission and sense of social obligation. These ethicality considerations are consistent with fundamental international ethical values (respect for autonomy, non-maleficence, beneficence and justice) [90]. Complex medical scenarios shared among multiple participants may potentially generate conflicts, wherefore common ethical values are crucial. These values may facilitate communication and enable carer staff and HCP participants to collaborate towards shared goals, e.g., the treatment plan, improving outcomes and potentially reducing the use of healthcare services [91].

### Intervention coherence/Perceived effectiveness

As far as intervention coherence was concerned, our findings surprisingly revealed considerable diversity in participants’ understandings of the main purpose of the intervention and how it worked or was expected to work. Fulfilling all participants’ individual goals is crucial for participant acceptance and to ensure an adequate SDM process [45, 51, 52]. Therefore, the documented diversity may potentially contribute to conflicts. Nevertheless, as all participants consistently expressed that they found that their (individual) main purpose had been fulfilled or would likely be fulfilled (perceived effectiveness), the diversity of goals appeared not to constitute a barrier to the overall acceptance of the intervention.

Thus, aligned ethicality and perceived effectiveness hold promise for future successful implementations.

### Affective attitude

In terms of affective attitude, all participants from all experience levels expressed positive feelings about the overall concept of shared MedCo. A positive attitude was enhanced when the participants enhanced their understanding of and experience with shared MedCo. The most prominent negative feelings were found among no experienced HCPs and carer staff participants. Both of these professionals voiced concerns about upsetting a stable medical equilibrium and the risk of adverse drug withdrawal events after medication changes or deprescribing. These concerns contributed to their initially negative attitude and an anticipated lack of acceptance of shared MedCo. This is in line with a pragmatic cluster-randomised trial (2024) that investigated deprescribing education among 3,012 adults aged 65 years and older who had cognitive impairment and were prescribed more than five chronic medications [92]. However, the trial found that no serious events, such as deaths, were associated with the deprescribing intervention during a four-month follow-up, and the total length of hospitalisation was reduced compared with that of the control group [92].

Another study (2018) found that deprescribing events and the risk of serious deprescribing harm are rare, especially when tapering and close monitoring are tailored to the individual patients [63]. In addition, this study found that prescribers, caregivers and patients all believed that good collaboration and a strong relationship with clear and open communication to achieve SDM were beneficial for positive deprescribing interactions [63]. The importance of good relationships and committing all participants across different contextual settings to the SDM process is elaborated on in a framework developed by researchers from Leeds University. The MIND-IT framework guides multiple participants in *Making Informed Decisions Individually and Together.* MIND-IT concluded that SDM occurs automatically if all involved participants, here from the healthcare and the social living setting, are committed through a comprehensive introduction and by fostering involvement, and if acceptance is achieved [51, 52].

These solutions equal the findings in our study, namely that deprescribing and language difference concerns can be countered by mechanisms such as performing shared MedCo with clear organisation and procedures, by fostering close relations and collaboration, and by adopting a patient-centred, structured SDM process with planning, tapering and close monitoring during and after medication withdrawal. Moreover, in our study, experienced carer staff and HCP participants did not express a feeling of deprescribing concern. Instead, they expressed a feeling of relatedness based on trust, respect and security as facilitators of participation in shared MedCo.

Additionally, several experienced carer staff and HCP participants noted that the participation of a clinical pharmacist, who conducted an overall medication review and was present during the SDM consultations, facilitated the negotiation and SDM while countering feelings of concern for medication adjusting or deprescribing. Rubio-Valera et al. (2014) demonstrated a broad range of skills and multiple roles for pharmacists in mental healthcare [39]. These included mechanisms such as medication management and counselling along with dissemination of information about the medication to prescribers [39]. The study also reported evidence of pharmacists participating in multidisciplinary teams, collaborating on medication therapy management and helping to reduce antipsychotic polypharmacy [39]. The presence of such a third healthcare professional who specialises in pharmacological treatment may counter physicians’ inherent respect for other physicians’ prescriptions and their inherent deprescribing concerns. In addition, the presence of a clinical pharmacist appeared to bolster physicians’ and carer staff’s confidence and willingness to make comprehensive shared medication decisions. Some similarities were found in a realist review investigating a deprescribing and medication review in multidisciplinary teams working with older people [31]. This study found that GPs were more likely to accept and discuss medication changes with their patients when a medication review with a clear plan and rationale was headed by a clinical pharmacist, although they harboured concerns about the potential risk of deprescribing medications for patients with frailty [31].

Overall, these findings suggest that the current shared MedCo set-up is suitable for counteracting anticipated deprescribing concerns and making shared decisions if all resident, carer staff and HCP participants met in person to collaborate supported by coordinators. Also, it may be assumed that a proper introduction and collaborative performance may facilitate security and ensure acceptance of shared MedCo.

### Burden/Opportunity cost

The burden/opportunity cost constructs identified multiple barriers and facilitators to the acceptance of shared MedCo. The elements identified acted as barriers when unresolved and as facilitators when addressed. The main topics included *practicalitie*s, *time perspective*,* PI* and *transferability*.

*Practical* support to the SDM process was noted by carer staff and HCP participants and recognised in the research literature for its impact on participants’ ability and competence to engage in the SDM process. Examples include mechanisms such as clear participant roles and work agreements, and the engagement of healthcare coordinators (such as a clinical pharmacist) to facilitate SDM between GPs and psychiatrists [31, 39, 42, 43], and a social living coordinator (like nurse coordinators managing logistics, overseeing schedules and residence health activities) to facilitate and support carer staff in resident involvement [40, 41, 45, 93]. Therefore, clear and practical solutions may facilitate the future acceptability of shared MedCo.

The *time perspective* was the most significant topic reported consistently across all interviews. The anticipated negative impact of time consumption reflects former research findings where participants were also concerned that the SDM intervention might be burdensome and time-consuming, and that the SDM intervention would be at the expense of other activities and patients [94, 95]. A systematic review by Légaré et al. (2008) identified limited time as a common barrier to SDM implementation among healthcare providers [96]. This was in line with an integrative review from 2020 [93]. As a solution, Hahlweg et al. (2023) further suggested that organisational resources, i.e. time and workforce, may need to be addressed at a higher policy level rather than being managed solely by individual participants or managers [97].

In contrast to these findings, all experienced participants in our study reported time savings when performing shared MedCo. The time and energy used were perceived as worthwhile to invest rather than burdensome, even during the intensive period around the shared residence consultations and even when new residents move into the facility with conditions that required close monitoring or assistance. The experienced carer staff, HCP and decision-maker participants reported long-term benefits of shared MedCo and considered shared MedCo worthwhile in terms of time spent and energy used, without sacrificing quality. This time perspective is supported by a systematic review (2023), which found that SDM and medical consultations do not necessarily lengthen consultation times; on the contrary, they may eventually save time [98]. As us, the researchers found that success requires initially coordinated efforts, participant support for SDM training, use of decision aids and alignment of consultations with patients’ needs. Furthermore, the researchers identified that awareness and acceptance of time challenges and involvement of higher-level decision-makers underpinned SDM success [98].

*PI* and SDM are feasible in psychiatric healthcare [47]. However, the nature of our study residents’ mental disorders may require carer staff support or advocacy in the SDM process. Thus, carer staff play a crucial role in PI by addressing concerns and uncertainties and improving residents’ ability to voice their views with HCPs [62, 93]. In our study, one striking finding was the differing statements made about PI burden voiced by the no experienced and experienced carer staff and HCP participants. Although expressing that PI was ethically correct, most no experienced participants doubted the feasibility of involving residents and anticipated that it would be burdensome. In contrast, experienced carer staff and HCP participants perceived PI as positive and beneficial. They emphasised that mechanisms such as leader support, building relationships, using a PI supporting tool and implementing organised MedCo procedures made PI possible, saved time and allowed them to gain energy without deducting time or resources from other tasks or patients. These experiences of giving voice to the residents is consistent with the literature; e.g., an integrative review (2020) that analysed 46 articles on SDM in serious mental illness [93] and a scoping review (2023) that screened 839 titles and abstracts to provide an overview of the opportunities to address employees’ voices in encounters with health care providers [62]. These studies found that barriers to implementing SDM and promoting PI included restricted patient decision-making abilities, limited staff motivation and interpersonal skills, impaired communication and troublesome interprofessional relationships [93]. However, the studies also found that HCPs specialised in severe mental disorders were more likely to perform PI owing to confidence gained from experience [93]. Furthermore, research suggests that staff may remain silent due to fear of repercussions and the hierarchical culture within healthcare, which seems to inhibit openness and lead to a lack of control, thereby negatively impacting behaviour [62]. When carer staff lack control or do not feel valued, this may lead to low engagement, high stress, and reduced intrinsic motivation [62, 99], thus reduced intervention acceptability [75]. Efforts at creating a safe, trustful, open and supportive environment in which carer staff feel empowered to express concerns seem crucial for promoting open communication in healthcare and PI [62]. Also, as experienced in our study, the comprehensive context-adapted PI healthcare tool seemed to allow residents to participate actively in healthcare decisions, carer staff to promote PI, clinical pharmacists to perform comprehensive resident-near medication reviews and health staff coordinators to understand residents’ needs and prevent language barriers between professional and layman language. However, it may initially be necessary to extend consultation times and ensure decision-maker support for the integration of interprofessional collaboration [62, 93].

The *transferability* of the Shared MedCo intervention has proven challenging despite its potential for residents who need multidisciplinary treatment. This raises concerns about its future applicability to other residential and community settings. Now, insights from the present study enhance the potential for transferring the intervention, as these findings can guide the implementation process. However, challenges may still exist.

In general, coordination of medication prescribed from multiple practitioners is crucial for residents and those living independently in a community setting. SDM and PI are central elements in the coordination approach and reflected in existing research evidence [100–103]. Thus, aspects of the Shared MedCo intervention, such as the core components (SDM, PI and organised MedCo), may benefit individuals living with severe mental disorders in a community-based care setting. However, circumstances such as context, available support, and participant characteristics may differ significantly. While residents depend on the carer staff, individuals living independently may have a higher functioning level, enabling more self-reliant and active engagement in the SDM process. In contrast, physical or cognitive challenges and limited support from relatives may hinder meaningful participation from the independent living individuals. Therefore, in general and despite the insights gained in the present study, those differences emphasis the need for sufficient time and resources to carefully adapt the intervention throughout the implementation process in a new residential or community context [104]. This adaptation should remain the core components of shared MedCo while adapting the approach to achieve a good fit between the intervention and the specific characteristics of the new target population and context. Such an approach is essential to ensure the continued relevance and effectiveness and thereby transferability of the shared MedCo intervention across different care settings [104].

### Self-efficacy

Low self-efficacy was most commonly reported among participants with no experience of shared MedCo, particularly among carer staff without a healthcare background, whereas experienced residents, carer staff, and HCPs generally expressed high levels of self-efficacy. The combination of a non-healthcare professional background, low self-efficacy in health communication and having responsibility for information exchange may potentially result in carer staff insecurity and silence. To address carer staff silence, Kepplinger (2023) emphasised the need for managers to support and empower carer staff to speak up and raise concerns about the residents, thereby improving healthcare and outcomes [62]. This is supported by Xu et al. (2019), who found that proper supervisor-subordinate congruencies are related to higher levels of psychological safety and, thus, a stronger voice among carer staff [65]. Furthermore, facilitators appeared to be supportive of all resident, carer staff and HCP participants’ competencies through SDM support (e.g., the PI healthcare tool), clear participant roles and task descriptions. These findings align with existing evidence showing that more knowledge and experience in the context of mental health services are associated with more SDM and PI [93]. Additionally, healthcare and social living coordinators (pharmacists and nurses) may streamline MedCo by enhancing HCPs’ self-efficacy and enabling SDM, i.e. in deprescribing medication, addressing language barriers and boosting carer staff’s self-efficacy to support or encourage that residents living with severe mental disorders engage in SDM [31–43, 93].

### Strengths and limitations

Using the TFA to evaluate the shared MedCo intervention was a *strength* for this study. This approach provided pre-defined constructs for addressing the complex phenomenon of acceptability. Another strength was the comprehensive empirical material, gained from multiple divergent participant levels, which provided advanced and contextually relevant insight and understanding of barriers and facilitators for transferring shared MedCo to other contextual settings. However, *limitations* were identified. In this study, the interviewer, a clinical pharmacist experienced in pharmacist-led psychiatric medication review, selected and invited participants, many of whom were known in advance. This raised concerns about selection bias and participant reluctance to criticise the intervention. To mitigate these issues, countermeasures were applied. These measures included reflecting on anticipated preconceptions before and after each interview [105], asking pertinent follow-up questions to enrich findings and diversifying participant selection based on the concept of information power to reduce blind spots [87]. Furthermore, the interviewer’s experience did not include practical healthcare and social living issues in residential contexts. Also, collaboration with co-authors affiliated with psychiatry, clinical pharmacists and nursing fields furthered transparency [106, 107]. Another limitation is the absence of representation from the municipal area, which might have nuanced our findings, particularly the economic aspects.

## Conclusion

Multiple barriers and facilitators at several levels of society (residents, carer staff, HCPs, decision-makers) and participants’ experience were identified for acceptance of shared MedCo. The main facilitating findings focus on SDM (through shared residence consultations), PI (by residents supported or advocated by carer staff) and organised MedCo (through involving pharmacist coordinators in the healthcare setting and nurse coordinators in the social living setting). Overall, knowledge and understanding of barriers to and facilitators of acceptance of shared MedCo is important for future implementers to counteract implementation issues, improve delivery and ensure transferability of the shared MedCo intervention.

Further research is needed to assess the intervention’s adaptability and effectiveness across settings. Future research should build on the identified barriers and facilitators to explore how the shared MedCo intervention can be adapted to meet local MedCo requirements within the contexts of new social psychiatric residences or in community-based care. Efforts should focus on developing a generic and adaptable shared MedCo intervention involving its core components (SDM, PI and organised MedCo), with particular emphasis on the residential carer staff group or relatives.

Moreover, it is important to recognise that what may seem as a simple intervention about conducting shared residence consultations turned out to be a complex intervention involving several interacting components, having a variety of outcomes and requiring new behaviours by those delivering and receiving the intervention. We therefore recommend that future implementers take a complex intervention approach and ensure sufficient time and space to conduct a feasibility or pilot test before large-scale implementation. Additionally, to better understand the context and the mechanisms of change, investigating the processes and effects of the shared MedCo intervention is essential. This method may support a proper introduction, adaptation and implementation approach, striving to achieve the acceptance of all participants affiliated with the context of residents living with severe mental disorders in social psychiatric residences.

## Supplementary Information

Supplementary Material 1: Consolidated Criteria for Reporting Qualitative Research.

Supplementary Material 2: Interview guide. Template.

Supplementary Material 3: Interview guide Residents. Template.

Supplementary Material 4: Comprehensive review of barriers and facilitators of acceptability of Shared MedCo.

## Data Availability

The datasets used and analysed during the current study are available from the corresponding author on reasonable request.

## Abbreviations

MedCo: Medication coordination
GP: General practitioner
HCPs: Healthcare professionals
SDM: Shared decision-making
PI: Patient involvement
TFA: Theoretical framework of acceptability
