# Supplementary material for: Acceptability of shared medication coordination in social psychiatric residence consultations: a qualitative interview study
Source: BMC Psychiatry. 2025 Sep 25;25:865. doi: 10.1186/s12888-025-07175-7 (PMC12465142; doi:10.1186/s12888-025-07175-7)
Supplement: Supplementary file 1 — Supplementary Material 1: Consolidated Criteria for Reporting Qualitative Research. [file 12888_2025_7175_MOESM1_ESM.docx]

## Additional file 1. COnsolidated criteria for REporting Qualitative research (COREQ)

**Table A.** Reporting according to COREQ (Tong et al., 2007).

| **Reporting criteria** |  | |
| --- | --- | --- |
| Domain 1: Research team and reflexivity | | |
| Personal characteristics |  |  |
| Interviewer/facilitator | Pg. 3 | |
| Credentials | Pg. 16 | |
| Occupation | Pg. 16 | |
| Gender | n.a. | |
| Experience and training | Pg. 3+16 | |
| Relationship with informants |  |  |
| Relationship established | Pg. 4 | |
| Participant knowledge of interviewer | Pg. 3 | |
| Interviewer characteristics | Pg. 3+16 | |
| Domain 2: Study design | | |
| Theoretical framework |  |  |
| Methodological orientation and theory | Pg. 3 | |
| Participant selection |  |  |
| Sampling | Pg. 4-5 | |
| Method of approach | Pg. 4-5 | |
| Sample size | Pg. 5 | |
| Non-participation | Pg. 5 | |
| Setting |  |  |
| Setting of data collection | Pg. 4 | |
| Presence of non-informants | Pg. 4 | |
| Description of sample | Table 1 | |
| Data collection |  |  |
| Interview guide | Pg. 4 | |
| Repeat interviews | Pg. 5 | |
| Audio/visual recording | Pg. 5 | |
| Field notes | Pg. 5 | |
| Duration | Pg. 5 | |
|  |  | |
| Data saturation | Pg. (5) | |
| Transcripts returned | Pg. 5 | |
| Domain 3: Analysis and findings |  |  |
| Data analysis |  |  |
| Number of data coders | Pg. 3 | |
| Description of coding tree | n.a. | |
| Derivation of themes | n.a. | |
| Software | Pg. 5 | |
| Participant checking | Pg. 5 | |
| Reporting |  |  |
| Quotations presented | Pg. 7-12 | |
| Data and findings consistent | All authors found consistency between presented data and the findings. | |
| Clarity of major themes | n.a. | |
| Clarity of minor themes | n.a. | |
