# Supplementary material for: Acceptability of shared medication coordination in social psychiatric residence consultations: a qualitative interview study
Source: BMC Psychiatry. 2025 Sep 25;25:865. doi: 10.1186/s12888-025-07175-7 (PMC12465142; doi:10.1186/s12888-025-07175-7)
Supplement: Supplementary file 2 — Supplementary Material 2: Interview guide. Template. [file 12888_2025_7175_MOESM2_ESM.doc]

| Briefing |  |  |
| --- | --- | --- |
| **Presentation of participant** | **- Tina**  - **Research Group** | Project manager, Pharmacist at the Hospital Pharmacy  Head-supervisor. Professor/nurse  Co-supervisor. Psychiatrist  Co-supervisor. Pharmacist |
|  |  |  |
| **Framing for conversation** |  | |
| Time frame | The conversation will last about 1 hour. | |
| Recording | The conversation will be recorded on a **recording device**. The recording is used to support my memory and will be included in my project work. | |
| Anonymisation | The conversation will be **treated as confidential and stored safely** at Aarhus University and will be deleted when the project is done.  Your statements will be **anonymized and mixed** with statements from other participants so they cannot be traced back to you. | |
| Role distribution | - **My role** is to understand and learn about your world as YOU experience it. I will therefore ask questions about your experiences.  I will take **notes** along the way in order to remember things that cannot be heard on the recorder.  - **Your role** is to answer based on your own experiences. | |
| Presentation | Please do not hesitate to **ask** if something is confusing or if somethings is not understandable during the conversation.  I remind you that you, of course, **participate** **voluntarily** and that you during the conversation always can decide to **withdraw your consent** if you wish to.  You can also **decide not to answer** certain questions. | |
| Process | We will see how the conversation shapes itself but overall:  - we start by talking about your concrete **experiences with medication coordination**  Later, the conversation shifts to be about:  - some **specific areas** that I would like to hear your experiences of  - how one can **transfer** the residence model to other residential institutions  - what would be your **dream scenario / advice** to other  How does that sound? | |
| Presentation of informant | Will you start out by **presenting** yourself?  Do not say your name but just who you are, what you do and why you are participating in this conversation. | |
| **Interview** |  |  |
| ***Theme*** | ***Main question*** | ***Experiences regarding Shared MedCo***  ***Assisting questions and guidance*** |
| Experiences with shared medication coordination and shared consultations | You are ………. and therefore involved in the coordination of the residents’ medicine at the social psychiatric residential institution.  In DK, **somatic** medicine is handled mainly by the patient’s general practitioner and their antipsychotic mainly by their psychiatrist.  Can you tell me – just overall – how you handle this?  Can you tell me – just overall – how you experience this? | - *Ask about relevant statements* |
|  | You told me that you have some shared meetings (consultations) between the residents and their general practitioner/psychiatrist. What do you **call** these meetings? | - *Do you know why you call them that?*   *(hereafter: "xmeeting")* |
|  | *What do you think the* ***main purpose*** *of these xmeetings are? 6*  *(Medicine optimisation / QoL)* | - *how does the model* ***achieve*** *this main purpose? 6*   *As I hear you describe it, the model consists of some overall measures (currently inclusion, pharmaceutical medication review, shared care meeting)*   - *how do the* ***individual*** *parts achieve this purpose? 6* - *what is* ***missing****? 6* - *how does the residence model match your* ***personal and moral core values*** *regarding residents with severe mental disorder? 3* |
|  | Now, I would like to go **back in time** to the last xmeeting you participated in.  Can you tell me in detail what you did before/during/after the xmeeting respectively? | ***Before:***   - *What did you do regarding:*   - *Initiation of the meeting*   - *Time planning*   - *Pre-testing*   - *Pre-interview with resident*   - *Resident support*   - *General practitioner/Psychiatrist*   - *Pharmacist*   - *Etc.* - *What did you do in the days leading up to the meeting?* - *What did you do on the day of the meeting?* - *What did you do immediately before the meeting?*   ***During:***   - *Who was present at the meeting?* - *What did you do during the meeting?*   ***After:***   - *What did you do immediately after the meeting?* - *What did you do in the days after the meeting?* |
|  | How did you **experience** xmeeting as a way to coordinate medicine? | 1. ***Feelings*** 2. ***Effort / workload*** 3. ***Ethics / Personal and moral core values*** 4. ***Coherence / meaning*** 5. ***Opportunity costs*** 6. ***Experienced effect (Main purpose)*** 7. ***Self-efficacy***   ***Worth the time and energy?***   - *What* ***effect*** *of xmeetings do you experience* ***between the xmeetings****?* - *Can you, with your experience, give a suggestion as to why this way of coordinating medicine* ***works for you?*** - *What do* ***others say*** *about this way of coordinating medicine?* - *How do you* ***compensate*** *for the* ***hard mental work*** *of having to be there for the residents (Staff companionship)?* - *How would you feel about* ***the resident/you*** *being the* ***mediator*** *at the meeting?*   *Would you say that words like xxx covers this?*  *Please give examples.* |
|  |  |  |
|  | In order to compare the residence model to places that do not have this model, I would like to talk about your experiences from **before** you performed it and was given the responsibility for the residence…  …. or: your experiences from other residential institutions that do not have xmeetings.  ……………………………………..  Now, I would like to talk about the time before this way of working. You were employed when the residense started doing the xmeetings.  If we once again go back in time, can you then tell me how you experienced the way the medicine was coordinated back then? | - What do you think **works well?** - What **challenges** are you experiencing here? - *Was/is there something that’s being a* ***bother*** *to you?* - *Can you elaborate? 2* - *How big an effort did/do you have to make to coordinate medicine here? 2* - *How did/do you experience that this way of doing medication coordination* ***makes sense*** *to/for you? 4* - *How did/do you experience that it* ***ties in*** *with other parts of your life / your resident’s medication treatment? 4* - *What* ***benefits****, profits, values* ***did/do you have to do without****? 5* - *What could/can you not do? 5* - *How did/does this way affect you emotionally? 1* - *Did you feel confident that you could/can display the behaviour required to participate in these meeting? 7* |
|  |  |  |
|  | It has now been 8-9 years since you started practising xmeetings. I would therefore like to take stock of it a bit together with you. | - What **challenges** do **still** exist? - What could counter these challenges? - Have you experienced any initiatives that you **have had to change/optimize afterwards**? Which and why?   Please give examples. |
|  |  |  |
|  | Now, I would like to talk to you about back when you started doing xmeetings. | - What was the reason for you starting it? - What was the reason for **doing it the way that you did**? (preliminary work, (incl. resident conversation and pharmaceutical medication review), xmeeting, follow-up) - What went **well/not so well**? - What challenges did you face during the **implementation itself**? - *How did this affect your feelings /* ***emotionally****? 1* - *Did you experience anything* ***bothersome*** *during the beginning? How? 2* - *How big an* ***effort*** *did you have to make to begin doing it? 2* - *What did you think* ***made sense*** *for you during the start-up phase? 4* - *How did you experience the work of trying to achieve* ***coherence*** *with the rest of the participants’ lives / medication treatment worked? 4* - *How many activities (benefits, profits, values) did you* ***have to give up*** *in the beginning? 5* - *What were you* ***not able to do*** *because you had to do the start-up process? 5* - *Would you, with your experience, say that it was* ***worth the time and energy?*** *Can you elaborate this?* |
|  |  |  |
|  | Is there something that, according to your experience, is important to **highlight** regarding this way of coordinating medicine? | - *What is* ***important*** *for you regarding this way of working?* - *Are there anything that* ***you do*** *that you see as* ***essential*** *for this whole set-up to work?* - *What do you imagine would happen* ***if*** *you* ***didn’t*** *do this?* |
|  |  |  |
|  |  |  |
| **Experiences with shared medication coordination, shared consultations, patient involvements, pharmacists and the residence** | Now, I would like to change it up and hear a bit more about what experience you have with some other short and more specific areas. | 1. ***Feelings*** 2. ***Effort / workload*** 3. ***Ethics / Personal and moral core values*** 4. ***Coherence / meaning*** 5. ***Opportunity costs*** 6. ***Experienced effect (Main purpose)*** 7. ***Self-efficacy***   ***Worth the time and energy?*** |
| Patients involvement | What experience(s) do you have regarding **inclusion of the resident** with severe mental disorder in the decision about own medical treatment? | - *Can you elaborate – please give some concrete examples…?* |
| Shared consultations | What experience(s) do you have regarding shared decision-making ex. during **shared meetings** between the residents and their general practitioner and psychiatrist. | - *Can you elaborate – please give some concrete examples…?* |
| Pharmacists | What experience(s) do you have with **pharmacists** that do medication review for this group of residents or just in general? | - *Can you elaborate – please give some concrete examples…?* |
| The reference residence model | What do you know about the social psychiatric residential institution (name of the residence)**?** | *Do you know about the way they work with medication coordination?*  *How does this way live up to your personal* ***moral and core values*** *regarding medication coordination?* |
|  | If you had to participate in this set-up, would there be anything that, according to your experience, is **important to highlight** about this way of coordinating medicine for it to work for you?  As in resident involvement, shared decision-making, pharmacists medication review? | - *In your experience, do you think that there is anything that is essential for this whole set-up to work for you?* - *What do you think will happen if this wasn’t the case?* |
|  |  |  |
|  |  |  |
| **Transfer to other residences** | Now, I would like you to imagine that you were given the job to implement something similar at another residential institution.   - How would you, with your experience, approach this? | - *In you experience, what elements would you say could help* ***achieve success*** *when starting this at another residential institution?* - *How would you make these elements come into play?* - *In you experience, what elements would you say could present* ***challenges*** *when it comes to starting this at another residential institution?* - *What would you* ***avoid*** *doing?* - *What would you* ***ask others to do*** */ avoid doing?*  1. ***Feelings*** 2. ***Effort / workload*** 3. ***Ethics / Personal and moral core values*** 4. ***Coherence / meaning*** 5. ***Opportunity costs*** 6. ***Experienced effect (Main purpose)*** 7. ***Self-efficacy***   ***Worth the time and energy?***   - *How would this affect the residents QoL*   *Would you say that words like xxx covers this?* |
|  |  |  |
| **Perspectives** | Now, I would like to dive all the way down a wishing well for the perfect world.  If there weren’t any outside restrictions at all; can you describe how the perfect medication coordination model could look like for you? | - *Why is this model perfect? Please give some examples.*  1. ***Feelings*** 2. ***Effort / workload*** 3. ***Ethics / Personal and moral core values*** 4. ***Coherence / meaning*** 5. ***Opportunity costs*** 6. ***Experienced effect (Main purpose)*** 7. ***Self-efficacy***   ***Worth the time and energy?***   - *How would this model affect your QoL / work satisfaction?*   *Would you say that words like xxx covers this*? |
|  | If you had to say in **three words** only what the medication coordination model gives you/could give you if it was implemented – what would they be? | - *Why these particular words?* |
|  |  |  |
|  |  |  |
| **End** | And finally, I would like to hear whether you think we have **covered everything** or if there is something I should have asked you about that I haven't? | - *Can you elaborate?* |
| **Debriefing** |  |  |
|  | Now we are about to be done with the conversation.  I hope it has been **fine for you** to participate in and optimally that you feel like you also **got something out of this**. |  |
| **Practical** | Your statements are very **valuable**. They will now be used to find the overarching **themes** that will describe what barriers and facilitators for acceptance are existing to **introduce the residence model to other places.** | Can I **contact you** if I have follow-up questions?  Can I contact you when I have found the overall **themes** so we can make sure that you **agree**? |
|  |  |  |
|  | Thank you so much for your help. | You are welcome to **contact me** / us you if you should have questions at some point. |
|  |  |  |

Follow-up questions in general:

- How? What happened?
- How did you experience it?
- What does it mean to/for you?
- Can you elaborate?
- Do you have more examples?
- You said xx, can you describe this?
- When you talk about xx, are you then thinking about yy or zz?
- Do you remember what they said?
- Because…? Had a feeling...?
- That experience… is it similar to other experiences you have had?
- You have said xx several times, what do you mean by xx?
- When you say xxx could that possible by xx
- Might you have misunderstood that?
