# Supplementary material for: Acceptability of shared medication coordination in social psychiatric residence consultations: a qualitative interview study
Source: BMC Psychiatry. 2025 Sep 25;25:865. doi: 10.1186/s12888-025-07175-7 (PMC12465142; doi:10.1186/s12888-025-07175-7)
Supplement: Supplementary file 3 — Supplementary Material 3: Interview guide Residents. Template. [file 12888_2025_7175_MOESM3_ESM.doc]

| Briefing |  |  |
| --- | --- | --- |
| **Presentation of participant** | **- Tina**  - **Research Group** | Project manager, Pharmacist at the Hospital pharmacy  Head-supervisor. Professor/nurse  Co-supervisor. Psychiatrist  Co-supervisor. Pharmacist |
|  |  |  |
| **Framing for conversation** | | |
| Timeframe | I have not chosen any specific length for this. My thoughts are to take the **amount of time that is fitting**. We stop whenever you think you have told everything or when I have found out what I need. | |
| Recording | The conversation will be recorded on this **recording device**. It is just so I will be able to remember what we have talked about and then I am going to use it in my project. | |
| Anonymisation | The recording will **only be used by me**, and I am going to **store it safely** at Aarhus University and will **delete it** when I am done with the project.  Everything you say **will be mixed** with what the other participants are saying so no one will be able to know what you have said. | |
| Role distribution | - **My role** is to understand and learn about your world as YOU experience it. I will therefore ask questions about your experiences.  I will take notes along the way in order to remember things that cannot be heard on the recorder.  - **Your role** is to answer based on your own experiences. | |
| Presentation | Please do not hesitate to **ask** if something is confusing or if somethings is not understandable during the conversation.  I remind you that you, of course, are here **voluntarily** and that you always can decide to stop if you wish to.    You also have the choice to **not answer** my questions if you do not want to. | |
| Presentation of informant | Will you start out by saying who you are and why you are here to talk to me?  Just do not say your name. | |
| **Interview** |  |  |
| ***Theme*** | ***Main question*** | ***Experiences regarding ShaPhaC.***  ***Assisting questions and guidance*** |
| Experiences with the reference residence model | You live here at the residence, and I know that you participate in the yearly health checks. |  |
|  | Now, I would like to go back in time to the last health check you participated in.  Do you remember this?  Can you try to tell me what you did before/during/after the meeting respectively? | *Before:*   - *What did you do regarding:*   - *Initiation of the meeting*   - *Time planning*   - *Pre-tests*   - *Pre-interview with residential supporter*   - *General practitioner/Psychiatrist*   - *etc.* - *What did you do in the days leading up to the meeting?* - *What did you do on the day of the meeting?* - *What did you do immediately before the meeting?*   *During:*   - *Who was present at the meeting?* - *What did you do during the meeting?*   *After:*   - *What did you do immediately after the meeting?* - *What did you do in the days after the meeting?* |
|  | *Why do you think that the / your residence are doing this meetings?*  *(Main purpose)? 6* | - *How does this live up to this main purpose? 6* - *What’s missing? 6* |
|  | How was it for you to talk about your medicine before, during and after the meeting?   - ***Why is it working*** *here?* - *Have you* ***talked to people*** *that do not live here about it? What do they say?* | 1. ***Feelings*** 2. ***Effort / workload*** 3. ***Ethics / Personal and moral core values*** 4. ***Coherence / meaning*** 5. ***Opportunity costs*** 6. ***Experienced effect (Main purpose)*** 7. ***Self-efficacy***   ***Worth the time and energy?***  *Do you believe that words like xxx covers this?*  Please give examples. |
|  | Is there anything that, according to your experience, is **important for you to do** to make it work for you? | - *What do you imagine will happen if you don’t do this?* |
|  |  |  |
|  |  |  |
| **Transfer to other residences** | If another residence wants to begin doing these meetings, what would you then say to them? | - ***Achieve success*** - ***Challenges*** - ***Avoid*** - ***Ask others to do*** */ avoid doing?*   ***1. Feelings***  ***2. Effort / workload***  ***3. Ethics / Personal and moral core values***  ***4. Coherence / meaning***  ***5. Opportunity costs***  ***6. Experienced effect (Main purpose)***  ***7. Self-efficacy***  ***Worth the time and energy?***  *Do you believe that words like xxx covers this?* |
|  |  |  |
|  |  |  |
| **Perspectives**  Only use if resident can handle it. | Now, I would like to dive all the way down a wishing well for the perfect world.  If there weren’t any outside restrictions at all; can you describe how the perfect medication coordination model could look like for you? | - *Why is this model perfect? Please give examples.*   ***1. Feelings***  ***2. Effort / workload***  ***3. Ethics / Personal and moral core values***  ***4. Coherence / meaning***  ***5. Opportunity costs***  ***6. Experienced effect (Main purpose)***  ***7. Self-efficacy***  ***Worth the time and energy?***   - *How will this model affect your QoL?*   *Would you say that words like xxx covers this?* |
|  | If you had to say in three words only what the reference-model gives you/could give you if it was implemented – what would they be? | - *Why these particular words?* |
|  |  |  |
|  |  |  |
| **End** | And finally, I would like to hear whether you think we have **covered everything** or if there is something I should have asked you about that I haven't? | - *Can you elaborate this?* |
| **Debriefing** |  |  |
|  | Now we are about to be done with the conversation.  I hope it has been **fine for you** to participate in and optimally that you feel like you also **got something out of this**. |  |
| **Practical** | Your statements are very **valuable**. They will now be used to find the overarching **themes** that will describe what barriers and facilitators are needed to **introduce the reference residence model to other places.** | *Can I contact you if I have follow-up questions?* |
|  |  |  |
|  | Thank you so much for your help. | You are welcome to **talk to** your carer staff if there should arise some things you would like to talk about.  If there is something I need to answer, your carer staff will contact us. |
|  |  |  |
